# Clinical significance of tumor-stroma ratio in head and neck cancer: a systematic review and meta-analysis

**DOI:** 10.1186/s12885-021-08222-8

**Published:** 2021-04-30

**Authors:** Alhadi Almangush, Rasheed Omobolaji Alabi, Giuseppe Troiano, Ricardo D. Coletta, Tuula Salo, Matti Pirinen, Antti A. Mäkitie, Ilmo Leivo

**Affiliations:** 1 Department of Pathology, University of Helsinki, Haartmaninkatu 3, P.O. Box 21, Helsinki, Finland; 2 Research Program in Systems Oncology, Faculty of Medicine, University of Helsinki, Helsinki, Finland; 3 Department of Oral and Maxillofacial Diseases, University of Helsinki, Helsinki, Finland; 4 Institute of Biomedicine, Pathology, University of Turku, Turku, Finland; 5 Faculty of Dentistry, Misurata University, Misurata, Libya; 6 Department of Industrial Digitalization, School of Technology and Innovations, University of Vaasa, Vaasa, Finland; 7 Department of Clinical and Experimental Medicine, Foggia University, Foggia, Italy; 8 Department of Oral Diagnosis, School of Dentistry, University of Campinas, Piracicaba, São Paulo Brazil; 9 Department of Pathology, University of Helsinki, Helsinki, Finland; 10 Cancer and Translational Medicine Research Unit, Medical Research Center Oulu, University of Oulu and Oulu University Hospital, Oulu, Finland; 11 Institute for Molecular Medicine Finland (FIMM), University of Helsinki, Helsinki, Finland; 12 Department of Public Health, University of Helsinki, Helsinki, Finland; 13 Department of Mathematics and Statistics, University of Helsinki, Helsinki, Finland; 14 Department of Otorhinolaryngology – Head and Neck Surgery, University of Helsinki and Helsinki University Hospital, Helsinki, Finland; 15 Division of Ear, Nose and Throat Diseases, Department of Clinical Sciences, Intervention and Technology, Karolinska Institutet and Karolinska University Hospital, Stockholm, Sweden; 16 Institute of Biomedicine, Pathology, University of Turku and Turku University Hospital, Turku, Finland

**Keywords:** Head and neck cancer, Tumor-stroma ratio, Tumor-stroma, Clinical relevance, Marker, Systematic review and meta-analysis

## Abstract

**Background:**

The clinical significance of tumor-stroma ratio (TSR) has been examined in many tumors. Here we systematically reviewed all studies that evaluated TSR in head and neck cancer.

**Methods:**

Four databases (Scopus, Medline, PubMed and Web of Science) were searched using the term tumo(u)r-stroma ratio. The preferred reporting items for systematic reviews and meta-analyses (PRISMA) were followed.

**Results:**

TSR was studied in nine studies of different subsites (including cohorts of nasopharyngeal, oral, laryngeal and pharyngeal carcinomas). In all studies, TSR was evaluated using hematoxylin and eosin staining. Classifying tumors based on TSR seems to allow for identification of high-risk cases. In oral cancer, specifically, our meta-analysis showed that TSR is significantly associated with both cancer-related mortality (HR 2.10, 95%CI 1.56–2.84) and disease-free survival (HR 1.84, 95%CI 1.38–2.46).

**Conclusions:**

The assessment of TSR has a promising prognostic value and can be implemented with minimum efforts in routine head and neck pathology.

## Background

Head and neck cancer constitutes a major health problem in many countries. Squamous cell carcinoma of the head and neck is the most common tumor in this anatomic region. About 60% of cancers in this region are diagnosed at an advanced stage (III-IV), which associates with poor prognosis [1]. Tissues of head and neck cancer, like other solid cancers, consist of both carcinoma cells and stroma cells. The current management of these tumors depends widely on TNM stage and WHO histologic grade, which sometimes fails to identify aggressive tumors especially at an early stage (I-II) of head and neck cancer. Of note, both TNM stage and WHO grade take into consideration cancer-related, but not stromal-related characteristics.

**Fig. 1:**
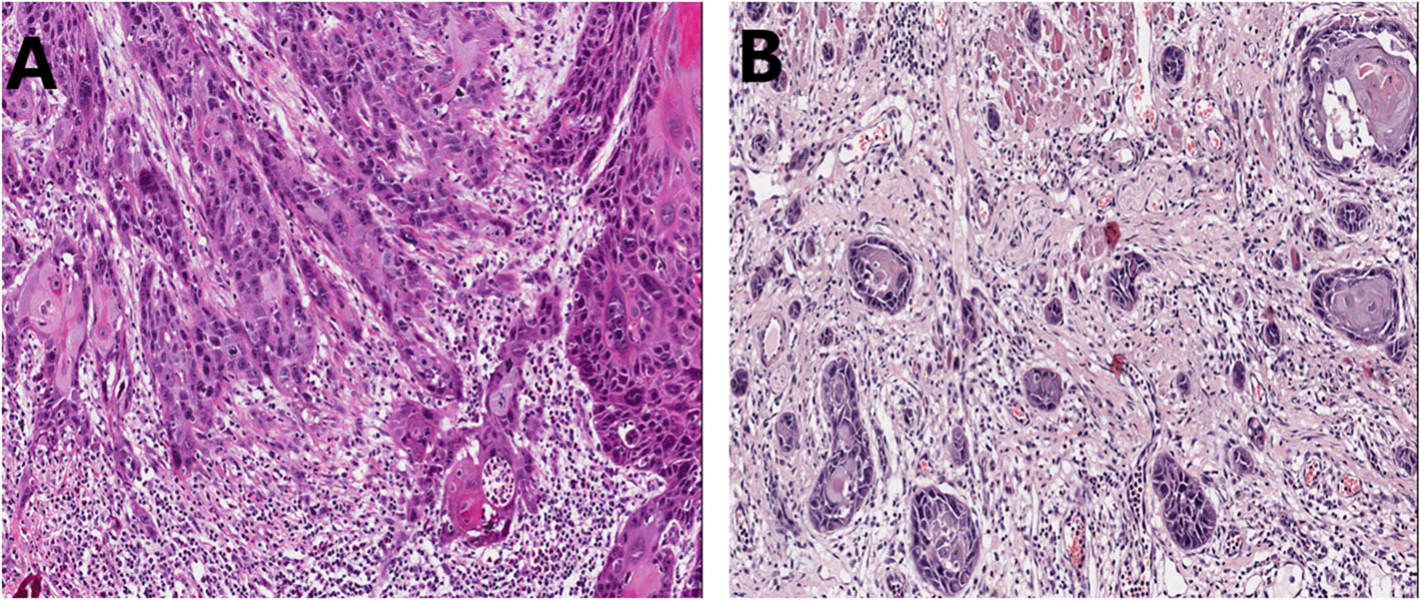
Hematoxylin and eosin-stained sections of oral squamous cell carcinoma. **a**: Tumor with a low proportion of stroma (< 50%). **b**: Tumor with a high proportion of stroma (≥ 50%) that associated with presence of tumor budding (i.e. small clusters of cancer cells) in a deeply invaded tumor

Analysis of prognostic markers expressed in tumor stroma has received more attention during the last decade. In recent studies the stroma of head and neck cancer has been analyzed mainly for specific molecules such as alpha-smooth muscle actin [2–5], which requires additional staining that is not routinely requested in daily clinical practice. For an ideal and practical prognostic marker, it is a great advantage that it can be assessed on routinely stained hematoxylin and eosin (HE) slides. Interestingly, tumor-stroma ratio (TSR), *defined* as the proportion of *tumor* tissue relative to surrounding *stromal tissue,* has been recently introduced as a valuable prognostic feature in many solid tumors [6–9]. In head and neck cancer, TSR (Fig. 1) has recently been introduced in different subsites as a promising prognostic feature [10–15]. However, in daily practice, the prognostic implication of TSR in patients with head and neck cancer remains less well recognized.

The aim of this study is to systematically search the literature for studies that have examined the significance of TSR in head and neck cancer. We also highlight the clinical relevance of TSR as an emerging prognostic marker that can be included in routine clinical practice. Recent recommendations [16] for the assessment of TSR are also briefly discussed.

## Methods

### Search strategy

Tumo(u)r-stroma ratio was used as a search word to retrieve any articles on this prognostic factor. The search included databases of Scopus, Medline, PubMed and Web of Science (all years until October 2020). Hits retrieved from this search were refined using the following terms: head and neck squamous cell carcinoma, head and neck cancer, oral cancer, oropharyngeal cancer, laryngeal cancer, nasopharyngeal cancer, hypopharyngeal cancer and sinonasal cancer. We included original reports, studies in English language and patient cohort studies. Therefore, review papers, studies in another language than English, and studies on animal samples were excluded.

Two independent researchers (A.A. & R.A.) conducted the search and screening processes which were followed by a discussion between the two researchers to ensure that all included studies met with the inclusion and exclusion criteria of the present study. The Preferred Reporting Items for Systematic Review and Meta-Analysis (PRISMA) were followed [17]. In addition, REporting recommendations for tumor MARKer prognostic studies (REMARK) guidelines [18] were considered when assessing the quality of the relevant studies. Further, Quality In Prognosis Studies (QUIPS) tool [19] was used to assess risk of bias in the relevant studies.

### Statistical analysis

The statistical software R (version 3.6.3) was used to run an inverse-variance weighted fixed-effect meta-analysis as implemented in the ‘meta’ package. Due to a small number of studies, we did not run a random-effect analysis. In addition to the meta-analyzed hazard ratios (HR), we also reported the estimated proportion of variation in effect sizes that was due to heterogeneity (*I*^*2*^) [20].

## Results

### Search results

**Fig. 2:**
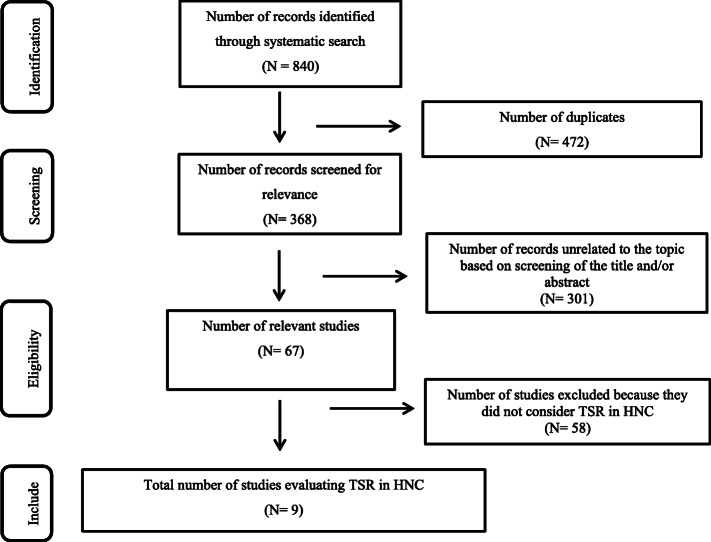
PRISMA flowchart of the search strategy and results

A total of 840 records were retrieved initially. After deleting duplicates and irrelevant papers, we found nine studies (Table 1) eligible to be included in this systematic review as they had analyzed tumor-stroma ratio (TSR) in head and neck cancer. The selection process of the articles is shown with PRISMA flowchart (Fig. 2). The included studies generally have low risks of bias (Table 2), and the quality of the included studies is generally acceptable to high.

**Table 1:** Summary of studies that reported tumor-stroma ratio (TSR) in head and neck cancer

First Author et al. Year (Country)	Tumor type	Stage	No. of cases	Stroma-rich cases (%)	Staining (Field)	Cutoff point	Main treatment	Main findings	Survival endpoint	Statistical values reported^**a**^
Zhang et al. 2014 (China) [11]	Nasopharyngeal	I-IV	93	45.16%	HE (×10)	50%	RT with or without CT	Stroma-rich tumors associated with poor prognosis	OS	HR 1.97 (1.10–3.55); *P* = 0.022 **HR 1.99 (1.06–3.74);** ***P*** **= 0.030**
									DFS	HR 2.06 (1.15–3.71); *P* = 0.015 **HR 1.92 (1.02–3.62);** ***P*** **= 0.042**
Niranjan et al. 2018 (India) [10]	Oral	I-III	60	30%	HE (×10)	50%	Surgery	TSR can be an adjuvant prognostic marker	OS	Survival rate was 77% in stroma-rich vs 95% in stroma-poor
									DFS	Survival rate was 44% in stroma-rich vs 69% in stroma-poor
Almangush et al. 2018 (Finland & Brazil) [12]	Oral tongue	I-II	311	28.6%	HE (×10)	50%	Surgery	Stroma-rich tumors associated with poor survival	DSS	HR 1.69 (1.02–2.79); *P* = 0.042 **HR 1.71 (1.02–2.86);** ***P*** **= 0.03**
									DFS	HR 1.67 (1.09–2.56); *P* = 0.02 **HR 1.81 (1.17–2.79);** ***P*** **= 0.008**
Karpathiou et al. 2019 (France) [15]	Laryngeal and pharyngeal	I-IV	266	22.6%	HE (×10)	50%	Surgery, and Neoadjuvant CT in 30% of cases	Stroma-rich tumors correlate with advanced stage and poor prognosis	OS	**(1.27–2.59);** ***P*** **= 0.001**
Karpathiou et al. 2020 (France) [21]	Laryngeal and pharyngeal	–	99	21.2%	HE (×10)	50%; also 30%	Surgery, and Neoadjuvant treatment	Stroma-rich tumors showed margin correlation with pretreatment measurements	NA	Correlation analysis of TSR with standardized uptake *P* = 0.07; and for metabolic tumor volume *P* = 0.1
Zhang et al. 2020 (China) [22]	Laryngeal	I-IV	51	48.6%	HE (×10)	50%	Surgery	TSR associated with poor survival. A significatint correlation between TSR and tumor budding was also reported	OS	*P* = 0.027
									RFS	*P* = 0.031
Dourado et al. 2020 (Brazil) [14]	Oral	I-IV	254	44.1%	HE (×10)	50%	Surgery, RT, CT	Stroma rich tumors associated with poor survival	DSS	HR 2.93 (1.89–4.52) *P* < 0.0001 **HR 3.58 (2.05–6.27);** ***P*** **< 0.0001**
									DFS	HR 2.29 (1.40–3.76); *P* = 0.001 **HR 2.05 (1.23–3.44);** ***P*** **= 0.006**
Bello et al. 2020 (Finland & Brazil) [23, 24]	Oral tongue	I-II	84	31%	HE (×10)	50%	Surgery	No significant difference in TSR between young and old patients	NA	NA
Mascitti et al. 2020 (Italy) [13]	Oral tongue	I-IV	211	19.43%	HE (×20)	50%	Surgery alone or with adjuvant RT and CT	Stroma rich tumors associated with poor survival in oral tongue cancer	DSS	**HR 1.68 (1.03–2.75);** ***P*** **= 0.036**
									OS	**HR 1.69 (0.99–2.56);** ***P*** **= 0.051**
									DFS	**HR 1.65 (0.92–2.96);** ***P*** **= 0.111**

*Abbreviations*: *CI* Confidence interval, *CT* Chemotherapy, *DFS* Disease-free survival, *DSS* Disease-specific survival, *HR* Hazard ratio, *HE* Hematoxylin and eosin, *NA* Not available, *RT* Radiotherapy, *OS* Overall survival, *RFS* Recurrence-free survival

^**a**^Values in bold are from multivariable analysis. Statistical values in parenthesis are 95%CI.

×10 objective was used for assessing the selected field

Almangush et al. 2018 [12] and Bello et al. 2020 [23] are overlapped

**Table 2:** Quality assessment of the relevant articles using Quality In Prognosis Studies (QUIPS) tool

Included studies	Bias Domains					
	Study participation	Study attrition	Prognostic factor measurement	Outcome measurement	Study confounding	Statistical analysis and reporting
Zhang et al. 2014 (China) [11]	Low risk	Low risk	Low risk	Low risk	Low risk	Low risk
Niranjan et al. 2018 (India) [10]	Low risk	Low risk	Low risk	Low risk	Low risk	Low risk
Almangush et al. 2018 (Finland & Brazil) [12]	Low risk	Low risk	Low risk	Low risk	Low risk	Low risk
Karpathiou et al. 2019 (France) [15]	Low risk	Low risk	Low risk	Low risk	Low risk	Low risk
Karpathiou et al. 2020 (France) [21]	Low risk	Low risk	Low risk	Low risk	Moderate risk	Low risk
Zhang et al. 2020 (China) [22]	Low risk	Low risk	Low risk	Low risk	Moderate risk	Low risk
Dourado et al. 2020 (Brazil) [14]	Low risk	Low risk	Low risk	Low risk	Low risk	Low risk
Bello et al. 2020 (Finland & Brazil) [23, 24]	Low risk	Low risk	Low risk	Moderate risk	Moderate risk	Moderate risk
Mascitti et al. 2020 (Italy) [13]	Low risk	Low risk	Low risk	Low risk	Low risk	Low risk

Of the nine studies included, one study considered TSR as an independent predictor for survival in nasopharyngeal cancer [11], five studies in oral squamous cell carcinoma [10, 12–14, 23], and three studies in laryngeal and pharyngeal squamous cell carcinomas [15, 21, 22]. A very good or perfect agreement between the observers in the assessment of TSR has been widely reported in the published studies [10–14], and it indicates a promising reproducibility. In the published studies [10–12, 14, 15], the samples were first scanned at low magnification to select the field with the highest amount of stroma and with tumor islands in all sides, and then this field is estimated at a higher magnification of × 100. It was noted that areas with high stroma (i.e. stroma-rich) were commonly near the area of deepest tumor infiltration [11, 12]. A cutoff point of 50% was widely used in the relevant studies (Table 1) to divide the cases into risk groups and the tumors were considered as either stroma-low (proportion of stroma < 50%) or stroma-high (proportion of stroma ≥50%).

### Prognostic significance of TSR in head and neck cancer

**Fig. 3:**
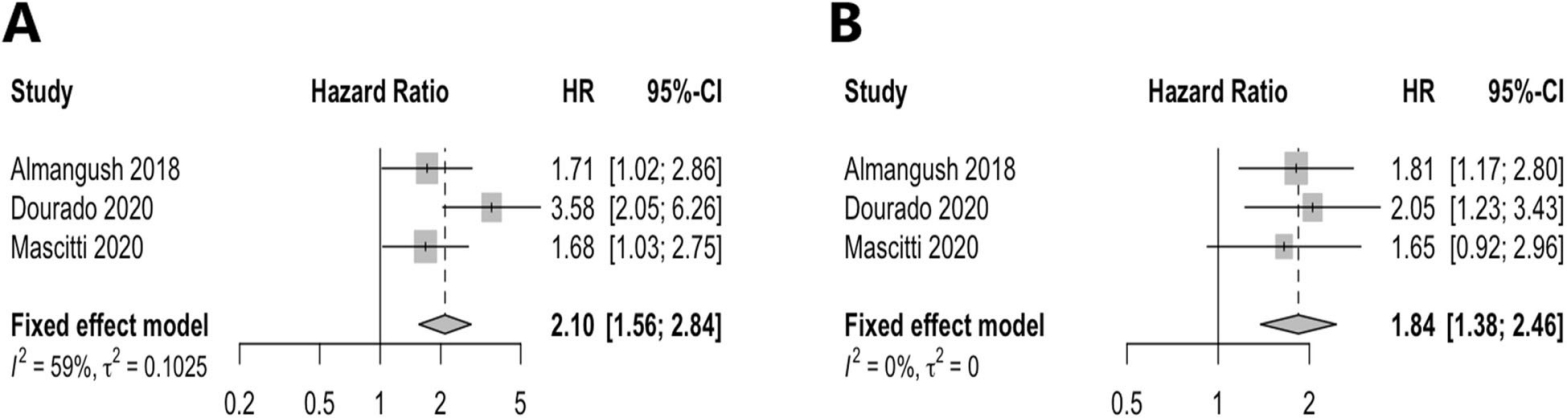
Forest plots for the meta-analysis of studies evaluated the prognostic value of tumor-stroma ratio in oral cancer. **a**: Disease-specific survival. **b**: Disease-free survival

Our meta-analysis of studies of oral cavity cancer (that was the most analyzed subsite), showed statistically significant association between TSR and survival (Fig. 3). We found that tumors with a high amount of stroma were associated with worse disease-specific survival with a hazard ratio (HR) of 2.10 and 95% confidence interval (CI) of 1.56 to 2.84 (Fig. 3a). In addition, such tumors were associated with worse disease-free survival (HR 1.84, 95%CI 1.38–2.46; Fig. 3b). In the meta-analysis, we observed some heterogeneity between the studies of disease-specific survival (*I*^2^ = 59%) but no heterogeneity (*I*^2^ = 0) between the studies of disease-free survival.

Although we were not able to conduct meta-analyses for the other subsites of head and neck (nasopharynx, pharynx, and larynx) due to the limited number of studies, the available studies reported promising predictive power of TSR as summarized in Table 1. For example, Zhang et al. [11] reported a promising prognostic value for TSR in nasopharyngeal cancer. In a cohort of laryngeal SCC [22], TSR showed a predictive power for overall survival and recurrence-free survival. In a cohort of laryngeal and pharyngeal squamous cell carcinoma, TSR showed a significant association with overall survival and response to chemotherapy [15]. In all these cohorts (nasopharyngeal, laryngeal and pharyngeal cancers), stroma-high tumors were associated with worse prognosis similar to our findings in the meta-analysis of the oral cavity subsite (Fig. 3).

### Association of TSR with clinicopathologic characteristics

Some of the published studies reported a significant association between TSR and clinicopathologic characteristics of head and neck cancer including tumor stage [15], perineurial invasion [12], deep invasion [10–12] and cell-in-cell invasion [25]. In addition, TSR showed correlation with tumor budding [15, 22] and poor lymphocytic host response [15]. One study reported a correlation between stroma-rich tumors and pretreatment measurements [21] including pre-treatment positron emission tomography/computed tomography measurements.

## Discussion

Tumor stroma can influence the clinical behavior of solid tumors [26]. The decision of multimodality treatment for head and neck cancer requires identifying cases at high risk of poor survival. Such identification is currently based on TNM staging and WHO grading. However, both criteria (i.e. staging and grading) rely on the characteristics of cancer cells without consideration of stromal features.

There is accumulated evidence indicating that tumor stroma is one of the key elements in cancer progression and it contains important cell types (e.g. cancer-associated fibroblasts) that can regulate cancer spread and influence the most fatal event, i.e. metastasis, through production of growth factors and extracellular matrix [27]. Tumors with high amounts of tumor stroma can benefit from stromal growth-promoting factors leading to a more aggressive behavior and worse survival [6].

Interaction of cancer cells with the surrounding cells of the tumor stroma has been widely studied. Epithelial to mesenchymal transition (EMT) is an important event during cancer invasion in which squamous cells acquire mesenchymal properties. EMT has been linked to invasive front of the tumor where cancer cells interface with stromal cells [28]. At the histologic level, high activity of tumor budding which reveals EMT (as it is associated with EMT-like changes [29, 30]) has been significantly correlated with high amount of tumor stroma in head and neck cancer [15, 22], and colorectal cancer as well [31]. This has been speculated as an impact of rich stroma which facilitates EMT [15]. Furthermore, cancer-associated fibroblasts (CAFs), which are abundant cells and constitute a main component of the tumor stroma, have been associated with induced EMT in head and neck cancer [32]. The relationship between stromal components (e.g. CAFs) and EMT was also reported in other cancers which emphasize the significance of tumor stroma in the process of EMT [33–35].

In general, it has been speculated that a high content of tumor stroma associated with worse survival indicates a high level of interaction between cancer cells and stromal cells [36]. Moreover, tumors with a high content of tumor stroma were associated with a poor lymphocytic response suggesting that a strong immune response can prevent the development of tumor stroma minimizing aggressiveness of the tumor [15]. Furthermore, many studies on head and neck cancer showed a significant correlation between a high content of tumor stroma with features of tumor aggressiveness (Fig. 1b) including perineurial invasion [12], depth of infiltration [10–12], cell-in-cell invasion [25], advanced stage and treatment resistance [15]. Of note, correlation between stroma-rich tumors and aggressive behavior has been reported in other cancer types as well [7, 36].

Assessment of stromal-related biomarkers and stromal characteristics has received increasing attention in recent years. In many cancers, TSR has been identified as an important prognostic factor [6–9]. Interestingly, a high level of agreement between observers has been reported in studies on head and neck cancer [10–14] as well as on other cancers [7–9]. In addition, assessment of TSR has been conducted using routine HE stained glass slides and can be performed within a few minutes [16, 22]. Aiming to standardize the method of assessment of TSR, van Pelt et al. [16] have recently introduced recommendations for the assessment of TSR. For scoring of TSR they recommended to consider the most deeply invasive part of the primary tumor. Areas with the highest amount of stroma are selected at low magnification (objective × 2.5 or × 5); then a stromal area which has tumor islands/cells present at all edges of the selected field is scored at a higher magnification (objective × 10). In cases of heterogeneity, the highest percentage of stroma is selected. Pelt et al. [16] further suggested using 50% as a cutoff value for dividing tumors as having low or high stromal content. Interestingly, these recommendations [16] were approved widely in the published studies of head and neck cancer using HE-stained sections, and therefore they can be proposed as a standard method for the evaluation of TSR in daily practice.

The current study has a few limitations including a relatively small number of studies on each subsite and the well-known heterogeneity in cancer behavior between the various subsites of head and neck. In addition, we were not able to include all studies on different subsites of head and neck cancers in one meta-analysis to avoid combining heterogenous cohorts which has been criticized previously [37, 38]. However, there is consistent evidence in all published studies indicating that TSR is a clinically relevant parameter in head and neck cancer. In all studies, head and neck cancers with a high amount of stromal content were associated with worse survival. This is in accordance with results on other cancers such as gastric, breast, cervical and colon carcinomas [6–9] suggesting a generalized nature of our conclusion. Assessment of TSR in good quality biopsies has been suggested recently for oral tongue cancer [24].

## Conclusions

The simplicity of assessment, the inter-observer reproducibility and the reliability of the prognostic value of TSR indicate that this cost-effective prognostic parameter could be implemented in routine diagnostics and clinical decision-making in head and neck cancer. Future research should consider the assessment of TSR in prospective studies of large cohorts of head and neck cancer. Furthermore, a recent study has introduced preoperative assessment of TSR in rectal cancer using magnetic resonance imaging [39]. Such methods for preoperative evaluation of TSR should be widely considered for head and neck cancer as well. Targeting the tumor stroma of tumors with high stromal content could form a new strategy in the management of head and neck cancer.

## Data Availability

The datasets used in this study are available from the corresponding author upon a reasonable request.

## Abbreviations

CAFs: Cancer-associated fibroblasts
CI: Confidence interval
EMT: Epithelial to mesenchymal transition
HR: Hazard ratio
HNC: Head and neck cancer
HE: Hematoxylin and eosin
QUIPS: Quality In Prognosis Studies
REMARK: REporting recommendations for tumor MARKer prognostic studies
PRISMA: Preferred Reporting Items for Systematic Review and Meta-Analysis
TSR: Tumor-stroma ratio
WHO: World Health Organization

## References

[1] Chow LQM (2020). Head and neck Cancer. N Engl J Med.

[2] Rahrotaban S, Mahdavi N, Abdollahi A, Yazdani F, Kaghazloo A, Derakhshan S (2019). Carcinoma-associated fibroblasts are a common finding in the microenvironment of HPV-positive Oropharyngeal squamous cell carcinoma. Appl Immunohistochem Mol Morphol.

[3] Ramos-Vega V, Venegas Rojas B, Donoso TW (2020). Immunohistochemical analysis of cancer-associated fibroblasts and podoplanin in head and neck cancer. Med Oral Patol Oral Cir Bucal.

[4] Almangush A, Bello IO, Keski-Santti H (2014). Depth of invasion, tumor budding, and worst pattern of invasion: prognostic indicators in early-stage oral tongue cancer. Head Neck.

[5] Dourado MR, Guerra ENS, Salo T, Lambert DW, Coletta RD (2018). Prognostic value of the immunohistochemical detection of cancer-associated fibroblasts in oral cancer: a systematic review and meta-analysis. J Oral Pathol Med.

[6] Kemi N, Eskuri M, Kauppila JH (2019). Tumour-stroma ratio and 5-year mortality in gastric adenocarcinoma: a systematic review and meta-analysis. Sci Rep.

[7] Vangangelt KMH, Green AR, Heemskerk IMF, Cohen D, Pelt GW, Sobral-Leite M, Schmidt MK, Putter H, Rakha EA, Tollenaar RAEM, Mesker WE (2020). The prognostic value of the tumor-stroma ratio is most discriminative in patients with grade III or triple-negative breast cancer. Int J Cancer.

[8] Zong L, Zhang Q, Kong Y, Yang F, Zhou Y, Yu S, Wu M, Chen J, Zhang Y, Xiang Y (2020). The tumor-stroma ratio is an independent predictor of survival in patients with 2018 FIGO stage IIIC squamous cell carcinoma of the cervix following primary radical surgery. Gynecol Oncol.

[9] Huijbers A, Tollenaar RA, Pelt v GW (2013). The proportion of tumor-stroma as a strong prognosticator for stage II and III colon cancer patients: validation in the VICTOR trial. Ann Oncol.

[10] Niranjan KC, Sarathy NA (2018). Prognostic impact of tumor-stroma ratio in oral squamous cell carcinoma - a pilot study. Ann Diagn Pathol.

[11] Zhang XL, Jiang C, Zhang ZX, Liu F, Zhang F, Cheng YF (2014). The tumor-stroma ratio is an independent predictor for survival in nasopharyngeal cancer. Oncol Res Treat.

[12] Almangush A, Heikkinen I, Bakhti N, Mäkinen LK, Kauppila JH, Pukkila M, Hagström J, Laranne J, Soini Y, Kowalski LP, Grénman R, Haglund C, Mäkitie AA, Coletta RD, Leivo I, Salo T (2018). Prognostic impact of tumour-stroma ratio in early-stage oral tongue cancers. Histopathology..

[13] Mascitti M, Zhurakivska K, Togni L, Caponio VCA, Almangush A, Balercia P, Balercia A, Rubini C, Lo Muzio L, Santarelli A, Troiano G (2020). Addition of the tumour-stroma ratio to the 8th edition American joint committee on Cancer staging system improves survival prediction for patients with oral tongue squamous cell carcinoma. Histopathology..

[14] Dourado MR, Miwa KYM, Hamada GB, Paranaíba LMR, Sawazaki-Calone Í, Domingueti CB, Ervolino de Oliveira C, Furlan ECB, Longo BC, Almangush A, Salo T, Coletta RD (2020). Prognostication for oral squamous cell carcinoma patients based on the tumour-stroma ratio and tumour budding. Histopathology..

[15] Karpathiou G, Vieville M, Gavid M, Camy F, Dumollard JM, Magné N, Froudarakis M, Prades JM, Peoc'h M (2019). Prognostic significance of tumor budding, tumor-stroma ratio, cell nests size, and stroma type in laryngeal and pharyngeal squamous cell carcinomas. Head Neck..

[16] van Pelt GW, Kjaer-Frifeldt S, van Krieken J (2018). Scoring the tumor-stroma ratio in colon cancer: procedure and recommendations. Virchows Arch.

[17] Moher D, Liberati A, Tetzlaff J, Altman DG, Group P (2009). Preferred reporting items for systematic reviews and meta-analyses: the PRISMA statement. BMJ..

[18] Altman DG, McShane LM, Sauerbrei W, Taube SE (2012). Reporting recommendations for tumor marker prognostic studies (REMARK): explanation and elaboration. BMC Med.

[19] Hayden JA, van der Windt DA, Cartwright JL, Cote P, Bombardier C (2013). Assessing bias in studies of prognostic factors. Ann Intern Med.

[20] Higgins JP, Thompson SG (2002). Quantifying heterogeneity in a meta-analysis. Stat Med.

[21] Karpathiou G, Gavid M, Prevot-Bitot N, Dhomps A, Dumollard JM, Vieville M, Lelonge Y, Prades JM, Froudarakis M, Peoc’h M (2020). Correlation between Semiquantitative metabolic parameters after PET/CT and histologic prognostic factors in laryngeal and pharyngeal carcinoma. Head Neck Pathol.

[22] Zhang H, Sheng X, Zhang S, Gu X (2020). The prognostic value of tumor budding in laryngeal squamous cell carcinoma. Transl Cancer Res.

[23] Bello IO, Almangush A, Heikkinen I, Haglund C, Coletta RD, Kowalski LP, Mäkitie AA, Nieminen P, Leivo I, Salo T (2020). Histological characteristics of early-stage oral tongue cancer in young versus older patients: a multicenter matched-pair analysis. Oral Dis.

[24] Bello IO, Wennerstrand PM, Suleymanova I, et al. Biopsy quality is essential for preoperative prognostication in oral tongue cancer. APMIS. 2021;129(3):118–27. 10.1111/apm.13104 33320967

[25] Almangush A, Makitie AA, Hagstrom J (2020). Cell-in-cell phenomenon associates with aggressive characteristics and cancer-related mortality in early oral tongue cancer. BMC Cancer.

[26] Valkenburg KC, de Groot AE, Pienta KJ (2018). Targeting the tumour stroma to improve cancer therapy. Nat Rev Clin Oncol.

[27] Sahai E, Astsaturov I, Cukierman E, DeNardo D, Egeblad M, Evans RM, Fearon D, Greten FR, Hingorani SR, Hunter T, Hynes RO, Jain RK, Janowitz T, Jorgensen C, Kimmelman AC, Kolonin MG, Maki RG, Powers RS, Puré E, Ramirez DC, Scherz-Shouval R, Sherman MH, Stewart S, Tlsty TD, Tuveson DA, Watt FM, Weaver V, Weeraratna AT, Werb Z (2020). A framework for advancing our understanding of cancer-associated fibroblasts. Nat Rev Cancer.

[28] Christofori G (2006). New signals from the invasive front. Nature..

[29] Grigore AD, Jolly MK, Jia D, Farach-Carson MC, Levine H. Tumor Budding: The Name is EMT. Partial EMT. J Clin Med. 2016;5(5):51. 10.3390/jcm5050051. 10.3390/jcm5050051 PMC4882480 27136592

[30] Attramadal CG, Kumar S, Boysen ME, Dhakal HP, Nesland JM, Bryne M (2015). Tumor budding, EMT and Cancer stem cells in T1-2/N0 Oral squamous cell carcinomas. Anticancer Res.

[31] van Wyk HC, Park JH, Edwards J, Horgan PG, McMillan DC, Going JJ (2016). The relationship between tumour budding, the tumour microenvironment and survival in patients with primary operable colorectal cancer. Br J Cancer.

[32] Wang Q, Zhang YC, Zhu LF, Pan L, Yu M, Shen WL, Li B, Zhang W, Liu LK (2019). Heat shock factor 1 in cancer-associated fibroblasts is a potential prognostic factor and drives progression of oral squamous cell carcinoma. Cancer Sci.

[33] Grunberg N, Pevsner-Fischer M, Goshen-Lago T, Diment J, Stein Y, Lavon H, Mayer S, Levi-Galibov O, Friedman G, Ofir-Birin Y, Syu LJ, Migliore C, Shimoni E, Stemmer SM, Brenner B, Dlugosz AA, Lyden D, Regev-Rudzki N, Ben-Aharon I, Scherz-Shouval R (2021). Cancer-associated fibroblasts promote aggressive gastric cancer phenotypes via heat shock factor 1-mediated secretion of extracellular vesicles. Cancer Res.

[34] Wang L, Saci A, Szabo PM, Chasalow SD, Castillo-Martin M, Domingo-Domenech J, Siefker-Radtke A, Sharma P, Sfakianos JP, Gong Y, Dominguez-Andres A, Oh WK, Mulholland D, Azrilevich A, Hu L, Cordon-Cardo C, Salmon H, Bhardwaj N, Zhu J, Galsky MD (2018). EMT- and stroma-related gene expression and resistance to PD-1 blockade in urothelial cancer. Nat Commun.

[35] Baulida J (2017). Epithelial-to-mesenchymal transition transcription factors in cancer-associated fibroblasts. Mol Oncol.

[36] van Pelt GW, Sandberg TP, Morreau H, Gelderblom H, van Krieken JHJM, Tollenaar RAEM, Mesker WE (2018). The tumour-stroma ratio in colon cancer: the biological role and its prognostic impact. Histopathology..

[37] Dayan D, Vered M (2013). Is immuno-expression of E-cadherin really a prognostic factor in head and neck cancer?. Oral Oncol.

[38] Coletta RD, Yeudall WA, Salo T. Grand Challenges in Oral Cancers. Front Oral Health*.* 2020. 10.3389/froh.2020.00003 PMC8757769 35047976

[39] Cai C, Hu T, Gong J, et al. Multiparametric MRI-based radiomics signature for preoperative estimation of tumor-stroma ratio in rectal cancer. Eur Radiol. 2021;31(5):3326–35. 10.1007/s00330-020-07403-6 33180166

